# Combining thermal hydrolysis and methylation-gas chromatography/mass spectrometry with X-ray photoelectron spectroscopy to characterise complex organic assemblages in geological material

**DOI:** 10.1016/j.mex.2019.10.034

**Published:** 2019-11-02

**Authors:** Graham Purvis, Naoko Sano, Cees van der Land, Anders Barlow, Elisa Lopez-Capel, Peter Cumpson, James Hood, Jake Sheriff, Neil Gray

**Affiliations:** a Earth, Ocean & Planetary Science Research, School of Natural and Environmental Sciences, Newcastle University, Newcastle upon Tyne, UK; b Ionoptika Ltd., Eastleigh, Hampshire, UK; c Materials Characterisation and Fabrication Platform (MCFP), Department of Chemical Engineering, The University of Melbourne, Melbourne, Victoria, Australia; d Mark Wainwright Analytical Centre, University of New South Wales, Sydney, New South Wales, Australia; e National ESCA and XPS Users’ Service (NEXUS), Newcastle University, Newcastle upon Tyne, UK

**Keywords:** Combining thermal hydrolysis and methylation-gas chromatography/mass spectrometry with X-ray photoelectron spectroscopy, XPS, GC/MS, Pyrolysis, TMAH, THM, Geological material, Organic geochemistry, Analysis

## Abstract

What follows is a method applicable generically to the analysis of low levels of organic matter that is embedded in either loose fine-grained or solid geological material. Initially, the range of organic compounds that could be detected in a geological sample using conventional pyrolysis chromatography/mass spectrometry was compared to the range that was detected using thermally assisted hydrolysis and methylation-gas chromatography/mass spectrometry (THM-GC/MS). This method was used to validate the synthetic components fitted to X-ray photoelectron spectroscopy (XPS) carbon spectra of the sample. Reciprocally, XPS analysis was able to identify the constituent carbon-carbon, carbon-oxygen and carbon-nitrogen bonds of the functional groups in the compounds identified by THM-GC/MS. The two independently derived outputs from the THM-GC/MS and the XPS techniques mutually validated the identification of organic compounds in our geological samples.

We describe in detail the improvements to:

• The preparation of geological samples for analysis by XPS.
• Measurements of organic material in geological samples using GC/MS.
• The use of THM-GC/MS and XPS data used together to characterise low levels of organic material in geological samples.

## Graphical abstract

**Figure:**
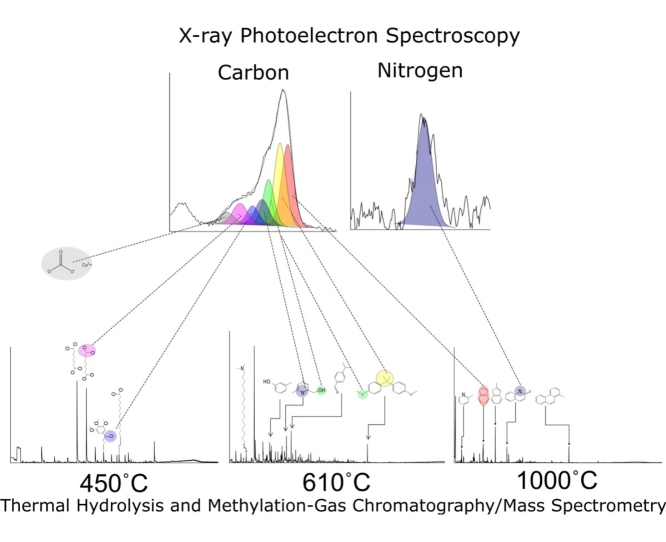

**Specification Table**

**Table:** 

Subject Area:	*Earth and Planetary Sciences*
More specific subject area:	*Organic Geochemistry*
Method name:	*Combining Thermal Hydrolysis and Methylation-Gas Chromatography/Mass Spectrometry with X-ray Photoelectron Spectroscopy*
Name and reference of original method:	*Purvis, Graham, Naoko Sano, Anders Barlow, Charles Cockell, Cees van der Land, Peter Cumpson and Neil Gray. "A stratigraphic comparison of the Organic Material in Submarine Basalts containing Microtubular Alteration Textures." Geobiology, 17(3), pp.281-293*
Resource availability:	www.openchrom.net/
	www.casaxps.com/berlin/

## Method details

### Background

Recently, eight samples of solid and loose fine-grained geological material were analysed to determine the provenance of the low to trace levels of indigenous organic material in these samples [1]. This analysis was conducted using a combination of thermal hydrolysis and methylation (THM) coupled gas chromatography/mass spectrometry (GC/MS), and X-ray photoelectron spectroscopy (XPS). GC/MS and XPS techniques each have their advantages and limitations when applied to the analysis of the complex organic chemistry occurring at trace levels in geological materials. Therefore, an analytical approach was developed that combined the results from these two instruments, which ameliorated the limitations of each of these instruments. This analytical approach permitted the comparison of the low levels of organic material in three solid volcaniclastic tuff samples along with three basalt control samples, a Miocene nano-fossil foraminiferal ooze, and a sample of fossilised charred higher plant material that had been buried in the tuff [1]. However, it was not possible to present the details of the methods in full, and the reasons for using these approaches in that original report.

#### Geological sample preparation and storage

Nitrile gloves were worn at all times and flamed forceps were used for handling samples according to the procedure described in [2]. Solid sections of rock were cut from the internal volume of parent samples to remove the outer 5–10 mm. The sections were trimmed into billets that were ca. 10 mm^2^ × 5 mm. All sample cutting was carried out on a Buehler Isomet 1000, cutting wheel (Buehler AG, Uzwil, Switzerland). Any moisture in the geological samples was removed by freeze-drying for 48 h in a Thermo Modulyo D0230 (Sciquip ltd., Shropshire, UK), for example from the calcareous foraminifer ooze studied in [1]. The geological samples were then cleaned using UV/O_3_ for 20 min, using a Jelight-144AX UV/O_3_ cleaner^tm^ (Jelight Co Inc., Irvine, CA, United States) [3,4] and subsequently stored in glass vials (sealed with UV/O_3_ cleaned Al foil under screw top lids).

#### Preparation of geological samples for GC/MS

The cleaned solid rock billets were hand milled in a UV/O_3_ cleaned agate pestle and mortar and then passed through a 150 μm grade, stainless steel screen that had been flamed to 750 °C. In contrast, the fine-grained material was used without any further preparation. These samples were weighed (ca. 10 mg) into clean quartz pyrolysis tubes, which were then plugged with extracted silica wool.

For conventional flash pyrolysis, the quartz tubes were immediately loaded into a CDS 1000 pyroprobe unit (CDS Analytical, USA). For THM-GC/MS analysis, 2 μl of 25%^v^/v TMAH (Sigma-Aldrich, UK) in double distilled water was added directly onto the samples in the quartz tube using a glass syringe before insertion into the pyroprobe unit. The maximum pyrolysis temperatures were set to either 450 °C, 610 °C and 1000 °C (see additional information). The pyrolysis temperature was maintained for 10 s (20 °C ms^−1^ temperature ramp) using a pulsed-mode open pyrolysis system, fitted with a platinum coil.

#### GC/MS operation

The pyroprobe unit was linked to the GC/MS via a CDS 1500 valved interface that was maintained at 320 °C. The products passed into an HP6890 gas chromatograph (GC) with a 60 m HP-5MS column (0.25 mm internal diameter, 0.25 um film thickness; J&W Scientific, USA). A helium carrier gas was used at a flow rate of 1 mL min^−1^. The GC was held at 50 °C for 1 min, and then the temperature was ramped from 50 °C to 310 °C at 5 °C min^−1^ and held at the final temperature for 12 min, for a total run time of 65 min. Product detection was carried out using an HP5972 series mass selective detector in full scan mode (50–650 u). Data acquisition was controlled by an HP kayak xa ChemStation computer.

#### Molecular identities of organic material in geological samples resolved using GC/MS

GC/MS Peak identification was conducted using the Openchrom software (www.openchron.net) using first derivative peak detection set to a ‘high’ threshold and a signal:noise ratio set to 5.0. The molecular identities of the peaks were then obtained with the Openchrom software, using a spectrum search of the NIST05 mass spectra library with reference to *Structure determination of organic compounds* [5]. These identities were then categorised into compound classes and tabulated according to the approach used in [6], and these identities were compared to the chemical data obtained by fitting synthetic peaks to the XPS high-resolution carbon spectra.

#### Preparation of geological samples for XPS

Solid rock billets were de-gassed for 48 h, prior to XPS analysis using a Thermo Modulyo D0230 freeze drier without using refrigeration, to reduce the time the XPS instrument required to obtain the ultra-high vacuum essential for the analysis. The de-gassed samples were placed directly onto the instrument analysis plate where they were immobilised by clips.

**Fig. 1:**
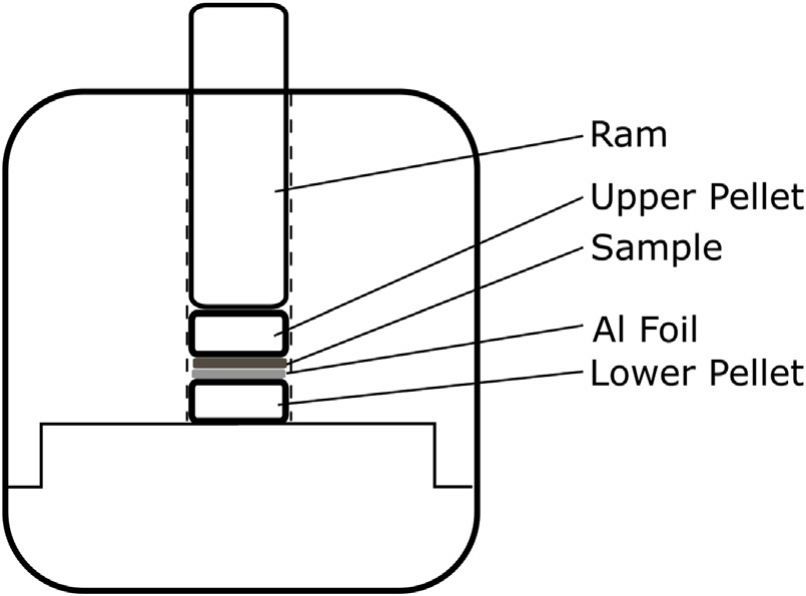
A diagrammatic representation of the apparatus used to compact loose fine-grained geological samples onto aluminium disks adapted from the apparatus used to form KBr disks for FTIR spectroscopy.

The fine-grained geological material had been previously immobilised by simply pressing samples on to double-sided adhesive tape on a sample holder. This material was, however, readily dislodged from the tape and drawn into the XPS instrument causing damage to the vacuum pump. This outcome was prevented by immobilising the fine-grained material by compaction onto aluminium foil. A 30 mm^2^ of Al foil was folded into an approximate 10 mm diameter disk and inserted into a pellet press apparatus used for the production of potassium bromide disks [7] the fine-grained geological sample was added to the top of the Al foil disk. The KBr pellet press was then assembled as normal (see Fig. 1) and pressure applied by a manual hydraulic press (Specac Ltd. Orpington, London, UK), using 1.5 t m^2^ of pressure. The compaction onto the aluminium disks conferred the additional advantage of reducing electrical charge build-up on the surfaces of the geological material. Alternately, any soft metal, such as indium, could be used in place of the aluminium. However, aluminium is less expensive and proved to be adequate for our investigations. The discs were attached to the XPS analysis plate using 3 M double-sided carbon tape.

#### XPS operation

The geological samples were placed into a K-Alpha XPS instrument (Thermoscientific Ltd., East Grinsted, UK). A gas cluster ion beam (GCIB) source was used to decontaminate the samples according to [4] using a Thermo Scientific^tm^ MAGCIS^tm^ gun (Thermoscientific Ltd., East Grinsted, UK) mounted within the XPS instrument. The GCIB was operated for 120 s at 4 keV generating a broad, semi-log distribution of argon cluster sizes centred on 1000 atoms per cluster at an input pressure of 4 bar. The beam current was stable around 20 nA ± 2 nA (as measured at the sample plate within the instrument). An ion beam raster of 1 mm × 2 mm was used, and the samples were immediately analysed without removal from the XPS instrument.

**Fig. 2:**
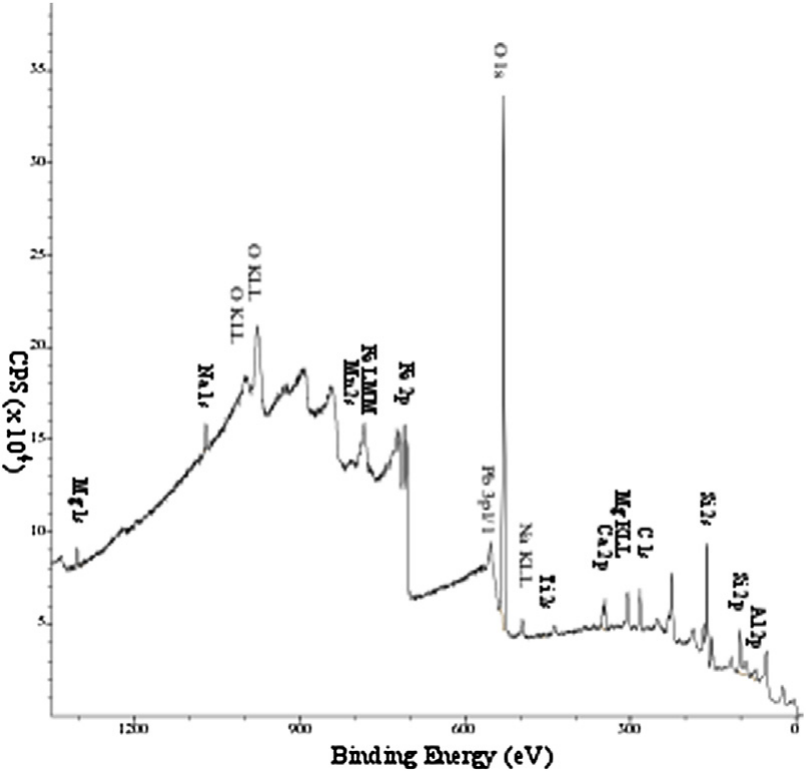
Example of a low-resolution broad survey XPS spectrum OJP.

XPS spectra were acquired using a monochromatic Al Kα X-ray source with a 1486.6 eV output energy with an X-ray spot size of 200 × 400 μm. Survey (wide energy, all elements) spectra were collected at 200 eV pass energy with a 1 eV step size (Fig. 2) whereas the high-resolution spectra were collected at a 40 eV pass energy with a 0.1 eV step size (Fig. 4a and b). Surface charge compensation was obtained with a low energy dual-beam electron/ion flood gun, and Schofield sensitivity factors were used. For future access, the XPS spectral data could be accessed in the internationally agreed, ISO14976 format. The carbon spectra were obtained from the C1s peak and nitrogen spectra from the N1s peak for both survey and high-resolution spectra.

#### The chemical identities of organic material in geological samples resolved using synthetic peak component fitting to XPS spectra

XPS data processing was carried out using CasaXPS software (CasaXPS ltd., Teignmouth, UK) and major peaks were selected for element identification using the Handbook of X-ray Photoelectron Spectroscopy [8]. The spectra were manually normalised and then summed to obtain mean carbon and nitrogen spectra, using CasaXPS. The peak regions with Shirley backgrounds were manually defined, and synthetic component fitting was conducted on resultant carbon and nitrogen high-resolution spectra.

The synthetic peak components were fitted to create a least-squared line of best-fit to the high-resolution spectra using a Marquardt linear regression analysis, which was part of the CasaXPS software package [9,10]. The chemical composition of the spectra was determined by comparing the binding energy position of the synthetic components to the known binding energies of chemical bonds in the La Surface and NIST XPS databases (www.lasurface.com and www.srdata.nist.gov/xps) and the references described in [8,11]. The number and positions of the synthetic components were informed by the data that was obtained from the THM-GC/MS analysis.

### Method assessment and validation

#### Comparison of conventional pyrolysis-gas chromatography/mass spectrometry and thermally assisted hydrolysis and methylation-gas chromatography/mass spectrometry to detect organic material in geological samples

**Fig. 3:**
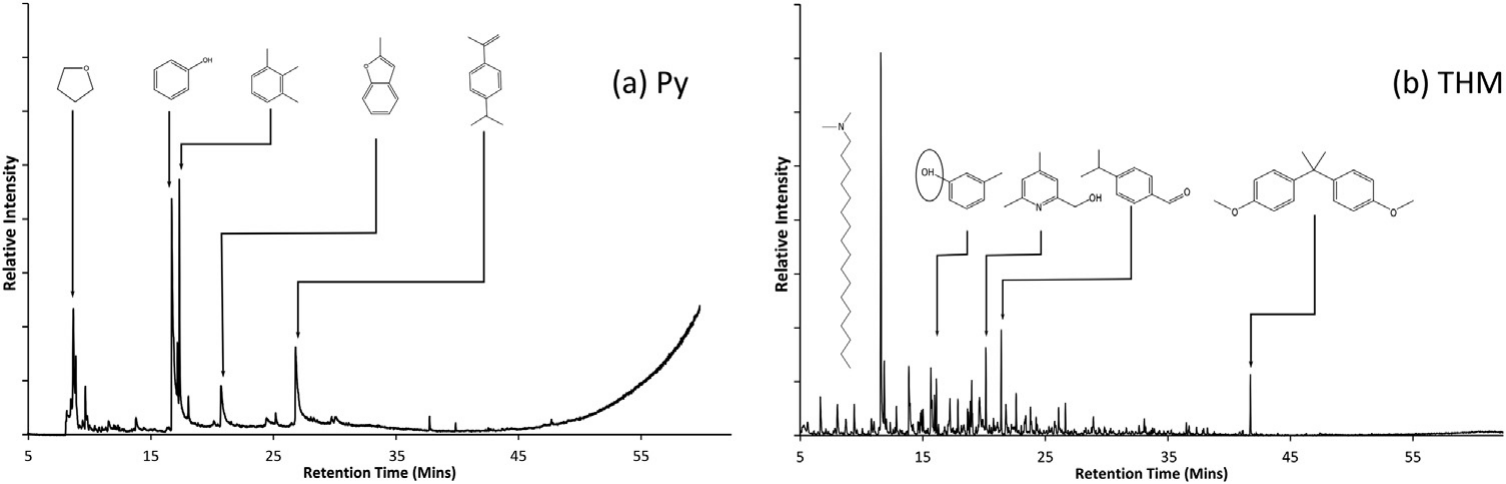
a and b. (a) The GC/MS total ion chromatograms (with some major peak molecular identity assignments) obtained from the OJP sample, using (a) conventional flash pyrolysis and (b) Thermal hydrolysis and methylation.

**Fig. 4:**
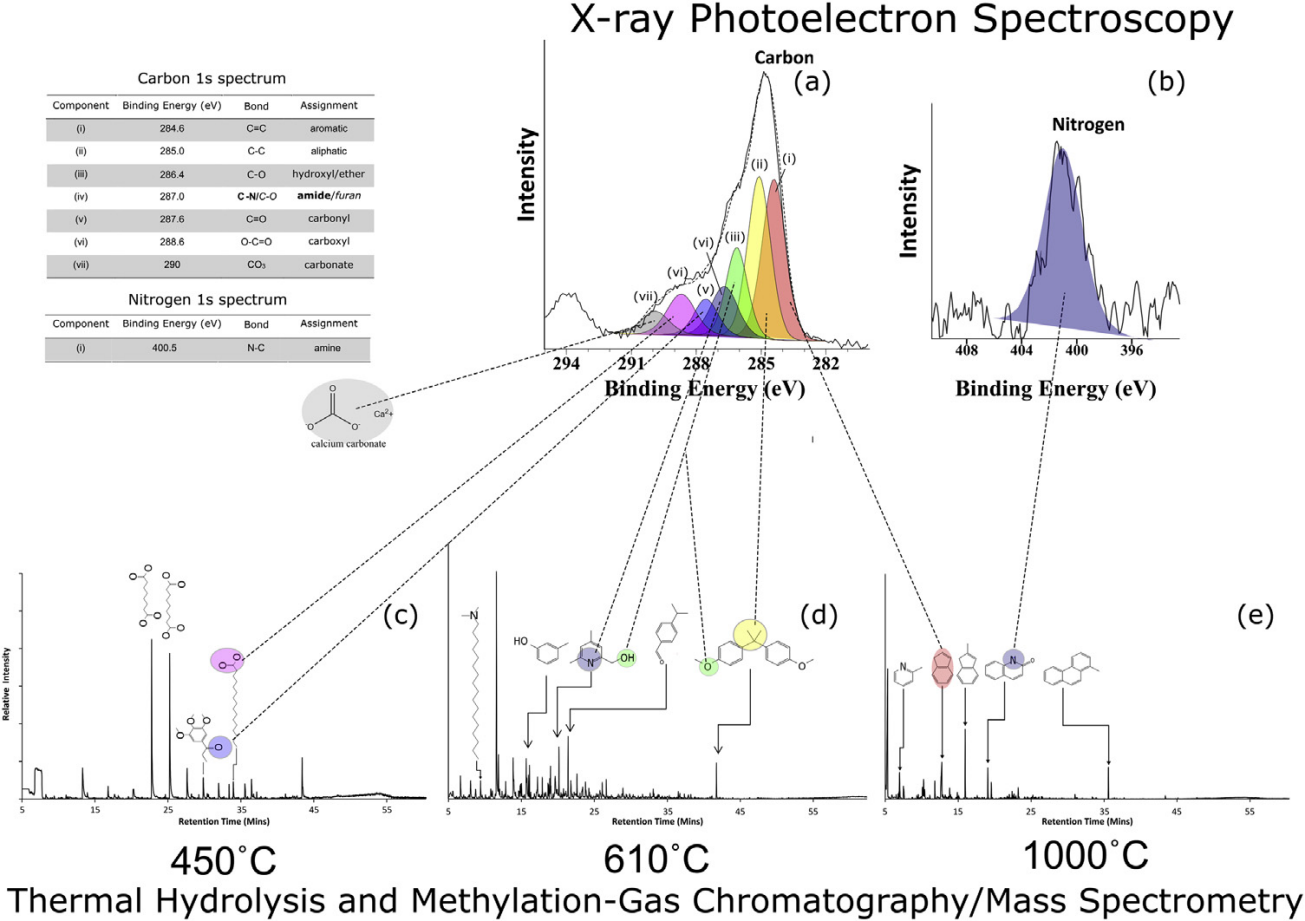
(a). OJP high-resolution XPS C1s spectra and (b) N1s spectra. The filled Gaussian peaks are the fitted synthetic components of the peak, providing an indication of the chemical states and the dashed line indicates the envelope used to obtain the best fit for the synthetic components. Examples of molecules with matching chemical functional groups are indicated in **(c)** THM-GC/MS at 450 °C (d) THM-GC/MS at 610 °C (e) THM-GC/MS at 1000 °C. The chemical structures are examples of the compounds identified by the NIST05 mass spectra library for the labelled peak.

Conventional flash pyrolysis-gas chromatography/mass spectrometry (py-GC/MS) was compared to flash pyrolysis in the presence of tetramethylammonium hydroxide (TMAH). This technique is sometimes called thermal hydrolysis and methylation-gas chromatography/mass spectrometry (THM-GC/MS). The pyrolysis temperature used in this experiment was 610 °C. Fig. 3a and b and Table 1 indicated that the addition of TMAH during pyrolysis (THM-GC/MS) increased the number of compounds that could be detected and identified by GC/MS. The compounds identified in the major peaks by reference to mass spectra in the NIST05 library possessed reverse match factors of between 0.7 and 0.9 indicating fair to good matches [[12], [13], [14]]. Major peaks observable in Fig. 4c–e were annotated with molecule structure diagrams as representatives of compound classes listed in Table 1. For comparison, this table also included the similarly derived analysis of the volcanic tephra JSC-1, using THM-GC/MS [6] and all the compound class assignments for both the major and minor peaks which were tabulated according to style described in [6].

**Table 1:** Chemical classes identified in the samples used in this investigation, using py-GC/MS (**Py**) and THM-GC/MS (**THM**) at 610 °C [1]. For comparison, the compounds identified in the JSC-1 using THM-GC/MS at 600 °C [5], N/D = No data, and includes other heterocycles. TOTAL = total number of compounds identified.

	Py	THM	JSC-1 [1]
Linear Carboxylic Acids	0	9	32
Linear Hydrocarbons	2	6	13
Alcohols	0	6	11
Amines/Amides	1	4	5
Aromatic hydrocarbons	7	70	87
Cyclic Hydrocarbons	0	9	N/D
Nitrogenous Heterocycles	0	9	30
other	0	3	7
TOTAL	10	116	185

#### XPS synthetic components and the corresponding functional groups in the compounds detected by THM-GC/MS analysis at three different pyrolysis temperatures

Table 2 presents an enumeration of the number of compounds assigned to different classes identified in the all peaks in the spectra from three different pyrolysis temperatures and that were matched with the mass spectra in the NIST05 mass spectral library, with reverse match factors of between 0.7 and 0.9 indicating fair to good matches [[12], [13], [14]]. Thermally labile compounds were detected using THM-GC/MS conducted at 450 °C, resulting in a high proportion (82.3%) of straight chain aliphatic compounds with carboxylic acid moieties, i.e. fatty acids (see Fig. 4c). These compounds are susceptible to thermal decomposition [15], and therefore fewer (7.8%) of these compounds were detected in the THM-GC/MS conducted at 610 °C and were absent in the THM-GC/MS conducted at 1000 °C. The results demonstrated that THM conducted at 610 °C yielded the highest number and most diverse range of organic compounds (see Fig. 4d). These included linear aliphatic, alkylated aromatic and heteroatomic alkylated aromatic molecules. Whereas, the compounds that were detected with THM-GC/MS conducted at 1000 °C were exclusively identified as single and multi-ring aromatic compounds (see Fig. 4e).

**Table 2:** Number of compounds assigned by different classes identified by the mass spectra of the peaks from three chemical compound classes that were identified in the OJP sample using THM-GC/MS with thermal extraction temperatures of 450 °C, (**THM_450_**), 610 °C (**THM_610_**) and 1000 °C (**THM_1000_**). Compound categories are indicative of the synthetic components assigned to the C1s XPS spectra that are annotated in Fig. 4a.

	THM_450_	THM_610_	THM_1000_
Linear Carboxylic Acids	14	9	0
Linear Hydrocarbons	0	6	0
Alcohols	0	6	0
Amines/Amides	1	4	0
Aromatic hydrocarbons	2	70	35
Cyclic Hydrocarbons	0	9	0
Nitrogenous Heterocycles	0	9	6
other	0	3	0
TOTAL	15	116	41

The high-resolution XPS spectra for carbon (Fig. 4a) from the OJP sample produced a complex peak shape, resulting from carbonaceous material with diverse chemical states that imply an array of organic compounds. Seven synthetic components were fitted to the OJP spectra (Fig. 4a i–vii), according to the description in the methods section, to produce a best fit, which is indicated by the envelope (dashed line). The number and position of each of the synthetic peaks that were chosen were informed by the information based on the NIST XPS and La Surface XPS databases and data provided in [11] and the molecular data that was obtained by THM-GC/MS, conducted at the three thermal extraction temperatures (Fig. 4c–e). The organic material in the OJP tuff sample was a complex mixture of compounds. However, six types of functional group were identified by THM-GC/MS across the range of three temperatures, and this complexity was reflected the six synthetic components that were associated with organic chemistry, which needed to be selected to fit the XPS C1s spectrum. Additionally, the proximity of aromatic and alkanes positions in the C1s spectrum (284.6 eV and 285.0 eV, respectively) could potentially have been resolved into a single component at ca. 284.8 eV to 284.9 eV, however, the identification of both aromatic and alkane moieties using THM-GC/MS implied that this part of the peak ought to be resolved into two synthetic components (Fig. 4a (i) and (ii)).

Fig. 4a shows that component (i) could be assigned to aromatic or alkene hydrocarbons but on balance is likely to be the former, based on the detection of n-alkylated aromatics by THM-GC/MS analysis conducted at 610 °C and 1000 °C. Component (ii) was consistent with aliphatic alkanes. These compounds correspond to the straight chain moieties of the fatty acids that were detected in the THM-GC/MS, particularly at 450 °C (thermal desorption) temperature. The C1s spectrum (Fig. 4a) components (iii) indicated C—O, bonds that were constant with both the hydroxyl and the ether functional groups. Component (iv) indicated either C—O bonds or C—N bonds, and the ambiguity of this assignment was resolved by the detection of nitrogen at 400.5 eV (Fig. 4b) indicating the presence of N—C bonds. Component (v) indicated C=O bonds and were consistent with ketones, and component (vi) was indicative of the carboxyl groups of straight chain fatty acids. Functional groups with bonds associated with these components were detected in the sample, by THM-GC/MS. The seventh C1s spectrum component (vii) was assigned to inorganic carbonates, most probably CO_3_, and was therefore not identifiable using GC/MS. In contrast to the C1s spectrum, the low nitrogen surface concentration resulted in an N1s XPS spectrum with a low signal to noise ratio. Therefore, any fine structure in the nitrogen spectrum was difficult to resolve (see Fig. 4b). A single synthetic component was fitted, which indicated the peak position was at ∼400.5 eV (Fig. 3b) to assist with the peak assignment. This peak position was consistent with N—C bonds, suggesting it was the reciprocal nitrogen signal of the Fig. 4a component (iv) of the C1s spectrum, which was assigned to C—N bonds. Reciprocally, the identification of the N—C bond (Fig. 4b (I)) and the C–N component (Fig. 4a (iv)) supported the identification of the pyrrole moiety in the OJP using THM-GC/MS (in Fig. 4d and e).

### Conclusion

A clearer indication of the range of organic compound classes in the samples was obtained using THM-GC/MS using a series of thermal extraction temperatures. However, pyrolysis temperatures of 610 °C provided the broadest range of compounds. The molecules that were detected and identified in this way allowed the synthetic components to be confidently fitted to the high-resolution XPS carbon spectrum of the OJP. XPS conducted measurements without the need for thermal extraction. Therefore, the XPS C1s scan was able to detect the full range of organic material irrespective of whether the organic material was capable of being desorbed from the host rock and being thermally mobilised, or not [16]. Furthermore, the XPS analysis mitigated any concerns regarding procedural artefacts resulting from thermally induced side reactions producing organic compounds that were not indigenous to the sample, e.g. [17,18]. The identification of specific chemical bonds by XPS provided greater confidence in the compounds classes that were identified, supporting the measurements made THM-GC/MS. This was particularly advantageous in the measurement of the organic material occurring in low concentrations in geological specimens, such as the samples used in [1], since thermal desorption of organic material from them has been reported to be a limiting factor, in such circumstances [16].

It may be possible to apply this method to organic material that is embedded in an inorganic matrix, such as in geological samples more generally. We speculate that this procedure may be useful in other disciplines for any analysis of low concentrations of organic material, which embedded in inorganic material, for example, the organic material embedded in pottery of archaeological or samples of paleobiological significance.

## Additional information

### The Example Geological Specimen Used to Demonstrate These Methods

Eight geological samples were analysed and described in [1]. A sample from that investigation was used here as an example to show the comparison between py-GC/MS and THM-GC/MS and the corroboration of the data from the THM-GC/MS and XPS instruments conducted in that investigation. The example specimen was a tuff that was obtained from Leg: 192, Hole: 1184A, core 13R core: 13R, interval: 145–148, of the Ocean Drilling Programme (http://iodp.tamu.edu/janusweb/coring_summaries/corelog.shtml) exploration of the Ontong Java Plateau. The 13R samples contained alteration textures that may have to indicate endolithic biological activity and the characteristics of organic matter embedded in these samples may provide evidence of the provenance of these alteration textures.

### Thermal hydrolysis and Methylation using Tetramethylammonium Hydroxide

Tetramethylammonium Hydroxide was used because its reaction with organic matter produces a more efficient thermal cleavage of chemical bonds, which permits lower pyrolysis temperatures to desorb and pyrolyse thermally labile compounds. This approach facilitated access to molecules that might otherwise be missed by conventional flash pyrolysis. Additionally, methylation converted polar compounds into non-polar compounds improving the transit of the released molecules through the GC column, particularly those with functional groups that are relevant in biomolecules, such as COOH, OH and NH_2_ [15].

### Thermal Hydrolysis and Methylation GC/MS Conducted at Three Pyrolysis Temperatures

The range of compounds detected by py-GC/MS is affected by the selected pyrolysis temperature, e.g. [[19], [20], [21], [22]]. Low thermal extraction temperatures (< 450 °C) will typically permit volatilisation and desorption of chemically unbound organic compounds but are usually not sufficiently high to induce pyrolysis. At higher temperatures (> 450 °C) organic compounds will still volatilise and desorb, but, will also usually undergo pyrolysis; a process which facilitates chemical bond scission through heating. Higher pyrolysis temperatures will cleave stronger chemical bonds resulting in higher levels of fragmentation in a consortium of organic compounds. However, excessively high pyrolysis temperatures may induce fragment decomposition and transformation of organic matter with the loss of molecular information, particularly for thermally labile organic compounds that may be of interest in biogeochemical studies. As a result, an intermediate pyrolysis temperature of ca. 600 °C is considered optimum for organic geochemistry, although in practice the Curie temperature, 610 °C, is commonly selected. e.g. [17,18,20,21,23]. Since a range of different pyrolysis temperatures was used, the term ‘thermal extraction’ is used to collectively refer to both ‘volatilisation’ and ‘pyrolysis’. In this current study, THM-GC/MS was conducted at 450 °C, 610 °C and 1000 °C.

### Synthetic Peak Component Fitting to High-Resolution X-Ray Photoelectron Spectra

The number and types of bonding environments of elements can be obtained by the sequential addition of a number of mathematical constructs called ‘synthetic components’ that are usually pre-defined as Gaussian shapes, and these are fitted to real high-resolution complex spectra (see graphical abstract). Since component fitting is a statistical algorithm, hypothetically, the repeated addition of components will eventually produce a series of synthetic components which perfectly match the XPS peak. However, over-fitting large numbers of components will result in the measurement of noise, meaning the model is unlikely to result in a realistic representation of the chemistry within a sample. This is particularly challenging with the complex organic geochemical compounds. Hence the requirement for compositional information from other techniques such as GC/MS in this case.

## Declaration of Competing Interest

None.
