# Supplementary material for: Combining thermal hydrolysis and methylation-gas chromatography/mass spectrometry with X-ray photoelectron spectroscopy to characterise complex organic assemblages in geological material
Source: MethodsX. 2019 Nov 2;6:2646–55. doi: 10.1016/j.mex.2019.10.034 (PMC6883294; doi:10.1016/j.mex.2019.10.034)
Supplement: Supplementary file 1 [file mmc1.docx]

# **Data Management Plan (Last Modified 24/10/19)**

# **Documentation and Metadata**

# Analytical Software code and Spectral data is stored on hard drive of the XPS hardware and in an encrypted folder on lead author’s personal portable hard drive. GC/MS data is stored on School of Natural and Environmental Sciences server and in an encrypted folder on Lead author’s personal portable hard drive versions would be named in keys of date and time.

# **Storage and Backup**

Data was recorded conforming to Newcastle University Research Data Policy. The raw data is documented in the form of *. VAMAS file formats and these may be read by ‘CasaXPS’ software and *.ocp file formats. GC/MS may be read by ‘Openchrom’ software. For future access, the XPS spectral data has been archived in the internationally agreed, ISO14976 format.

the results and analysis are stored on the University RAS: J Drive. Backups of the cited academic literature are stored on lead author’s H: Drive (RAS) in Endnote *.xml file format
